# Interaction of gibberellin and other hormones in almond anthers: phenotypic and physiological changes and transcriptomic reprogramming

**DOI:** 10.1038/s41438-021-00527-w

**Published:** 2021-05-01

**Authors:** Peng Li, Jia Tian, Changkui Guo, Shuping Luo, Jiang Li

**Affiliations:** 1 College of Forestry and Horticulture, Xinjiang Agricultural University, Urumqi, 830052 China; 2 Research Institute of Pomology, Chinese Academy of Agricultural Sciences, Xingcheng, 125100 China; 3 School of Agriculture and Food Science, Zhejiang Agriculture and Forestry University, Hangzhou, 311300 China

**Keywords:** Reproductive biology, Gibberellins, Transcriptomics

## Abstract

Low temperature causes anther dysfunction, severe pollen sterility and, ultimately, major yield losses in crop plants. Previous studies have shown that the gibberellic acid (GA) metabolic pathway plays an important role in this process by regulating tapetum function and pollen development. However, the interaction mechanism of GA with other hormones mediating anther development is still unclear. Herein, we collected and analyzed almond (*Amygdalus communis* L.) anthers at the meiosis, tetrad, 1-nucleus, and mature 2-nucleus stages. The growth rate per 1000 anthers exhibited a significant positive correlation with the total bioactive GA compound content, and the levels of all bioactive GA compounds were highest in the 1-nucleus pollen stage. GA_3_ treatment experiments indicated that exogenous GA_3_ increased the levels of indole-3-acetic acid (IAA), *trans*-zeatin (*t*Z), and jasmonic acid (JA) and decreased the levels of salicylic acid (SA) and abscisic acid (ABA); moreover, GA_3_ improved pollen viability and quantities under cold conditions, whereas PP_333_ (paclobutrazol, an inhibitor of GA biosynthesis) was antagonistic with GA_3_ in controlling anther development. RNA-seq and qRT-PCR results showed that GA played an important role in anther development by regulating the expression of other phytohormone pathway genes, dehydration-responsive element-binding/C-repeat binding factor (DREB1/CBF)-mediated signaling genes, and anther development pathway genes. Our results reveal the novel finding that GA interacts with other hormones to balance anther development under normal- and low-temperature conditions in almond.

## Introduction

A suitable temperature is a necessary condition for the survival of organisms, but the adaptability of various organisms to changes in temperature differs considerably^1^. A significant difference between plants and other multicellular complex organisms is that plants live in a fixed state. Therefore, throughout their life cycle, plants are forced to endure a variety of adverse environmental conditions, among which low temperature is one of the main limiting factors affecting the natural distribution and crop yield of plants^2^. In subtropical to frigid regions, overwintering plants have evolved strategies to adapt to the low-temperature environment. Deciduous fruit trees need to experience a certain period of low temperature to undergo natural dormancy to normally blossom and bear fruit^3^. However, too low of a temperature will lead to the freezing of branches, reductions in fruit yield and fruit quality, and even death^4^.

After a chilling injury occurs, plant hormone levels are adjusted to reduce the impact on growth and development. As an important hormone regulating anther development, gibberellic acid (GA) plays an important role in resisting chilling injury. Previous studies have shown that bioactive GAs (GA_1_, GA_3_, GA_4_, and GA_7_) function in pollen exine formation and programmed cell death of tapetal cells in *Arabidopsis*^5^, rice^6^, tomato^7^, and peach^8^. In addition, cold treatment damages anthers in the young microspore stage and induces severe pollen sterility and yield reduction, which can be partially recovered by spraying with GA^9^. However, how and why GA functions in almond reproductive processes under normal and low temperatures remain unclear. Previous research has shown that the B_3_ domain-containing transcription factor FUS3 plays a role in controlling the GA-ABA balance^10^ and that GA and abscisic acid (ABA) act antagonistically to control *Arabidopsis* development. ABA-induced by low temperature is involved in the degradation of the tapetum^11^, and the accumulation of ABA affects sugar transport, resulting in rice pollen sterility^12^. In addition, GA interacts with other hormones, including jasmonic acid (JA)^13^, indole-3-acetic acid (IAA)^14^, cytokinins^15^, and salicylic acid (SA)^16^, to participate in plant development. JA promotes the growth of stems and anthers through *MYB* genes^17^, and 24 GA-responsive genes and 82 JA-responsive genes work together to regulate the development of rice anthers^18^. IAA is required for the development of diploid microsporocyte cells, and IAA affects GA_1_ biosynthesis by maintaining *PsGA3ox1* gene transcript levels^19^; furthermore, GA promotes IAA accumulation through the GA-DELLA-LEC1 (LEAFY COTYLEDON 1) signaling cascade^20^. In the rice (*Oryza sativa*) meristem, the KNOTTED1-like homeobox (KNOX) protein activates cytokinin biosynthesis by upregulating the *isopentenyl transferase* (*IPT*) gene and subsequently reduces the expression of *GA2ox*^20^. In tomato, SA, GA, and antioxidant enzymes can interact with one another and protect tomato fruit from chilling injury and oxidative damage^21^.

Almond (*Amygdalus communis* L.) is considered the most important tree nut crop worldwide^22^. However, its nut yield is approximately 500 kg per hectare in Xinjiang, China, which is only one-quarter of that in California, USA. The low yield of almonds is mainly caused by the extremely low-temperature climate in the winter and early spring. In China and other Eurasian countries, almond trees require chilling to develop fruiting buds^21^. From autumn to winter, a reduction in gibberellin content under short photoperiod and chilling temperatures affects almond bud development and dormancy, and reproductive tissues become resistant to freezing^23^. However, extremely low temperatures below −20 °C occurring in the winter can damage reproductive tissues, including by having effects on microsporogenesis, which is susceptible to low temperatures, leading to severe reductions in nut yields^24^. Under increasing gibberellin levels and temperatures, anther reproductive tissue develops quickly and becomes less resistant to frost^25^. To increase the almond yield and reduce the losses caused by freezing damage, a number of experiments have been performed and have shown that different almond varieties exhibit differences in resistance^26^. In response to low temperature, the expression of GA-related genes is altered in almond anthers^4^; in the regulation of almond anthesis, *C-repeat binding factor* (*CBF*) and *gibberellin 20 oxidase* (*GA20OX*), which are involved in gibberellin biosynthesis, work together to regulate flower bud break. Spraying with GA_3_ either alone or in combination with potassium nitrate (KNO_3_) or benzyl adenine (BA) during the winter can induce earlier bud burst and increase the percentage of floral budburst, fruit set, and branch induction^27^.

The Chinese almond industry is limited by cold winds from Siberia, and the breeding of new almond varieties with high quality, high yields, and cold resistance is the only way to further develop the almond industry in Xinjiang. Extremely low temperatures reduce bioactive GA levels and induce severe pollen sterility, which can be restored by exogenous GA_3_. As a woody plant, the almond has evolved many specific strategies to respond to low temperatures. Whether GA functions in the mediation of almond anther development under cold conditions and how GA interacts with other hormones during anther development remain unknown. In this study, we analyzed the phenotypic and physiological changes in almond anthers and explored GA-regulated networks during anther development using RNA-seq and qRT-PCR. We obtained the novel finding that GA interacts with other hormones to control anther development and screened out key genes related to high-quality resistant pollen that may be used to breed new almond varieties.

## Results

### Anther development is associated with phytohormone levels

To explore the relationships between anther development and the levels of endogenous GAs, we first analyzed microspore development in almond. The results showed that the growth rate per 1000 anthers was 0.10 mg/d in ZP1 (“Zhipi” almond at the meiosis stage in the field), 0.21 mg/d in ZP2 (“Zhipi” at the tetrad stage in the field), and 3.38 mg/d in ZP3 (“Zhipi” at the 1-nucleus stage in the field) and then fell to 0.69 mg/d in ZP4 (“Zhipi” at the mature 2-nucleus stage in the field) (Table 1), indicating that rapid growth of anthers occurs from the tetrad stage to the 1-nucleus stage.

**Table 1:** Weight and growth rate per 1000 anthers

	ZP1	ZP2	ZP3	ZP4	PP_333_-treated	CK	GA_3_-treated
Weight of 1000 anthers (mg FW)	29.30 ± 0.31 Gg	36.20 ± 0.25 Ff	154.60 ± 1.04 Ee	297.90 ± 3.17Aa	164.00 ± 0.51 Dd	175.4 ± 0.95 Cc	188.4 ± 1.14 Bb
The growth rate of 1000 anthers							
(mg/d FW)	0.10 ± 0.001 Gg	0.21 ± 0.002 Ff	3.38 ± 0.03 Aa	0.69 ± 0.02 Ee	1.88 ± 0.04 Dd	2.16 ± 0.03 Cc	2.74 ± 0.04 Bb

Different letters indicate significant differences between treatments or stages according to one-way ANOVA at *P* < 0.01 (capital letter) and *P* < 0.05 (lowercase letter). FW fresh weight

**Fig. 1:**
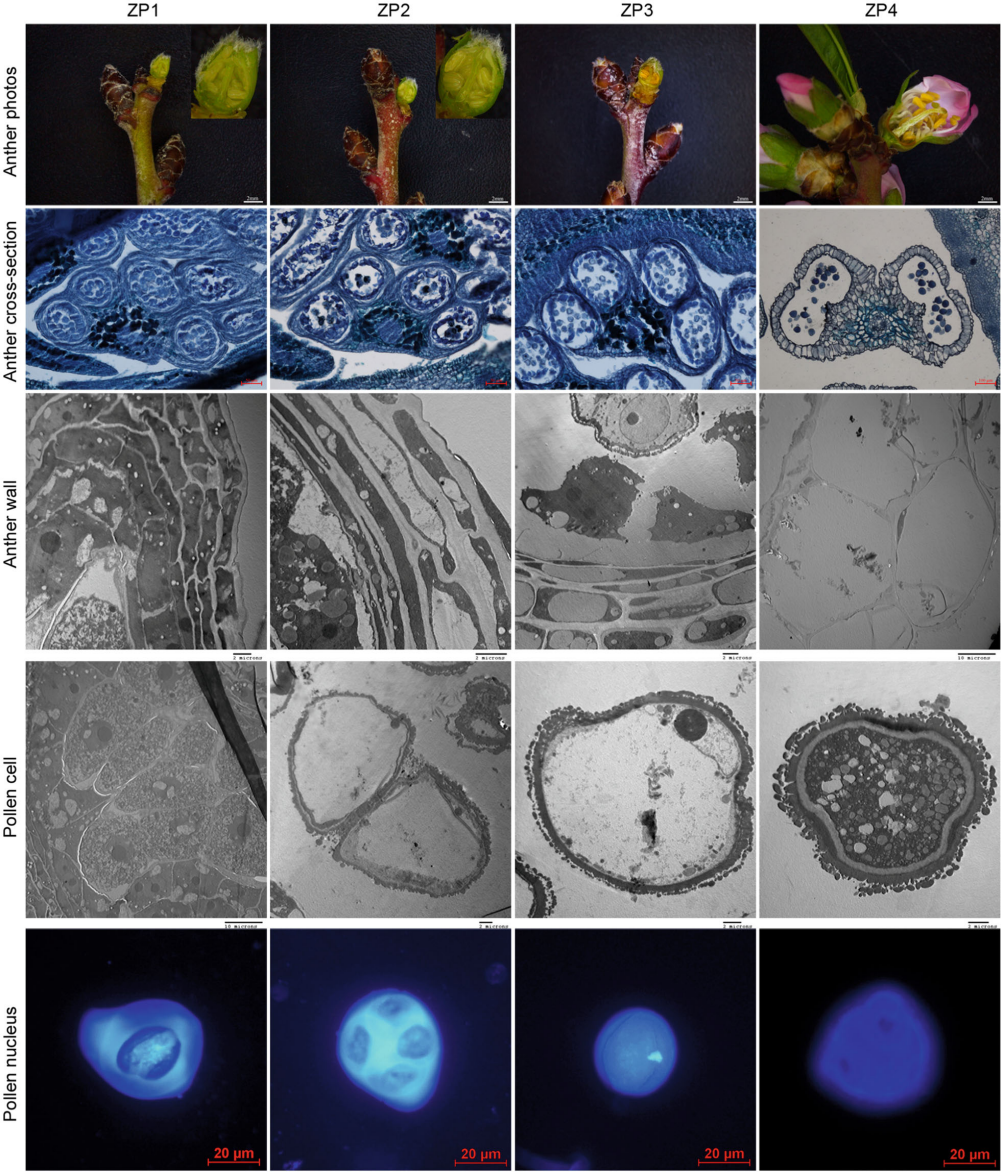
Analyses of anther development in almond. ZP1, meiosis stage in the field; ZP2, tetrad stage in the field; ZP3, 1-nucleus stage in the field; and ZP4, mature 2-nucleus stage in the field. Anthers were photographed using a camera; the sections were photographed using microscopes; the anther wall and pollen cells were observed using SEM, and the pollen nucleus was stained with DAPI and photographed under a microscope

The ZP1–ZP3 anthers exhibited five cell layers, whereas the ZP4 anthers presented three cell layers. The middle-layer cells were small and exhibited small vacuoles in the ZP1 anthers, began to undergo degradation in the ZP2 anthers, and were almost completely degraded in the ZP4 anthers. The multinucleate secretory tapetum exhibited a clear boundary and small vacuoles in the ZP1 anthers; the vacuoles in this structure became larger in the ZP2 and ZP3 anthers, and the multinucleate secretory tapetum had completely disappeared in the ZP4 anthers. The meiotic cells in the ZP1 anthers contained many fat droplets, starch granules, and small vacuoles, and some meiotic cells contained replicating DNA. The tetrad cells in the ZP2 anthers contained larger vacuoles, were covered by sporopollenin, and showed close positioning of the nucleus to the pollen wall. The 1-nucleus pollen in the ZP3 anthers presented a small number of starch granules and fat droplets and was covered by sporopollenin. Finally, the mature 2-nucleus pollen of the ZP4 anthers contained more fat droplets and starch granules but no vacuoles and was covered by sporopollenin (Fig. 1).

GA and other hormones function together in anther development in almonds. We sampled the anthers at four developmental stages to test the levels of hormones using high-performance liquid chromatography with tandem mass spectrometry (HPLC-MS). The results indicated that the levels of GA_4_, which mainly functions in anther development, increased significantly with pollen development. GA_7_ was detected at higher levels in the ZP1 and ZP2 anthers. Accordingly, their precursors, namely, GA_24_ and GA_9_, were present at relatively low levels. Other bioactive GAs, such as GA_1_ and GA_3_, were detected in only the ZP3 anthers, whereas their precursors, including GA_44_ and GA_20_, showed high levels. These results indicated that bioactive GAs mainly functioned at the 1-nucleus pollen stage and that the growth rate per 1,000 anthers exhibited a significant positive correlation with the total bioactive GA compound content (Table 2). We also examined the levels of other hormones, including ABA, JA, *trans*-zeatin (*t*Z), IAA, and SA. The levels of ABA, the antagonistic hormone of GA, decreased from ZP1 to ZP3 but rapidly increased in ZP4 anthers. However, the levels of JA, *t*Z, IAA, and SA were much higher in the ZP3 anthers and exhibited a significant decline in the ZP4 anthers (Table 2). These results suggested that GA might exert an effect similar to that of JA, *t*Z, IAA, and SA and opposite to that of ABA in regulating almond anther development.

**Table 2:** Plant hormone levels (ng/g FW)

	ZP1	ZP2	ZP3	ZP4	PP_333_-treated	CK	GA_3_-treated
GA_1_	ND Dd	ND Dd	0.390 ± 0.004 Bb	ND Dd	0.028 ± 0.000 Cc	0.818 ± 0.004 Aa	ND Dd
GA_3_	ND Ee	ND Bb	0.021 ± 0.001 Aa	ND Ee	ND Ee	0.010 ± 0.001 Dd	0.015 ± 0.001 Cc
GA_4_	0.095 ± 0.000 Cc	0.068 ± 0.001 Dd	0.152 ± 0.002 Bb	0.164 ± 0.002 Aa	ND Gg	0.004 ± 0.000 Ff	0.058 ± 0.001 Ee
GA_7_	ND Ff	0.122 ± 0.001 Dd	0.905 ± 0.012 Bb	ND Ff	0.055 ± 0.001 Ee	0.155 ± 0.003 Cc	3.011 ± 0.027 Aa
GA_24_	0.146 ± 0.001 Bb	0.065 ± 0.001 Ff	0.114 ± 0.001 Cc	0.277 ± 0.002 Aa	0.087 ± 0.001 Ee	0.093 ± 0.001 Dd	0.027 ± 0.001 Gg
GA_9_	0.280 ± 0.003 Aa	0.065 ± 0.001 Cc	0.095 ± 0.002 Bb	0.051 ± 0.000 Dd	ND Gg	0.023 ± 0.001 Ee	0.008 ± 0.000 Ff
GA_44_	11.603 ± 0.020 Cc	12.401 ± 0.049 Bb	21.210 ± 0.092 Aa	4.177 ± 0.012 Gg	5.616 ± 0.011 Ff	6.726 ± 0.015 Ee	7.577 ± 0.023 Dd
GA_20_	0.001 ± 0.000 Gg	0.028 ± 0.001 Ff	0.379 ± 0.001 Bb	0.171 ± 0.001 Ee	0.233 ± 0.001 Cc	0.625 ± .003 Aa	0.195 ± 0.003 Dd
ABA	2.536 ± 0.021 Dd	1.025 ± 0.006 Ee	0.275 ± 0.004 Ff	17.641 ± 0.134 Aa	8.167 ± 0.049 Cc	8.603 ± 0.053 Bb	0.350 ± 0.004 Ff
IAA	0.488 ± 0.001 Dd	0.378 ± 0.001 Ee	0.806 ± 0.011 Aa	0.168 ± 0.002 Ff	0.532 ± 0.002 Cc	0.528 ± 0.002 Cc	0.754 ± 0.004 Bb
JA	120.40 ± 1.35 Dd	38.16 ± 0.26 Ee	355.29 ± 5.98 Aa	8.47 ± 0.07 Ff	236.24 ± 1.59 Cc	238.53 ± 1.62 Cc	284.39 ± 1.92 Bb
SA	1.241 ± 0.004 Ee	1.201 ± 0.004 Ee	2.504 ± 0.019 Dd	0.861 ± 0.005 Ff	6.101 ± 0.022 Bb	6.225 ± 0.017 Aa	5.481 ± 0.034 Cc
tZ	0.549 ± 0.003 Ee	0.577 ± 0.003 Ee	0.753 ± 0.009 Dd	0.296 ± 0.004 Ff	5.795 ± 0.046 Cc	6.385 ± 0.033 Bb	9.010 ± 0.049 Aa

Different letters indicate significant differences between treatments or stages according to one-way ANOVA at *P* < 0.01 (capital letter) and *P* < 0.05 (lowercase letter). *FW* fresh weight, *ND* not detected

### GA affects anther development and the levels of other hormones in anthers

**Fig. 2:**
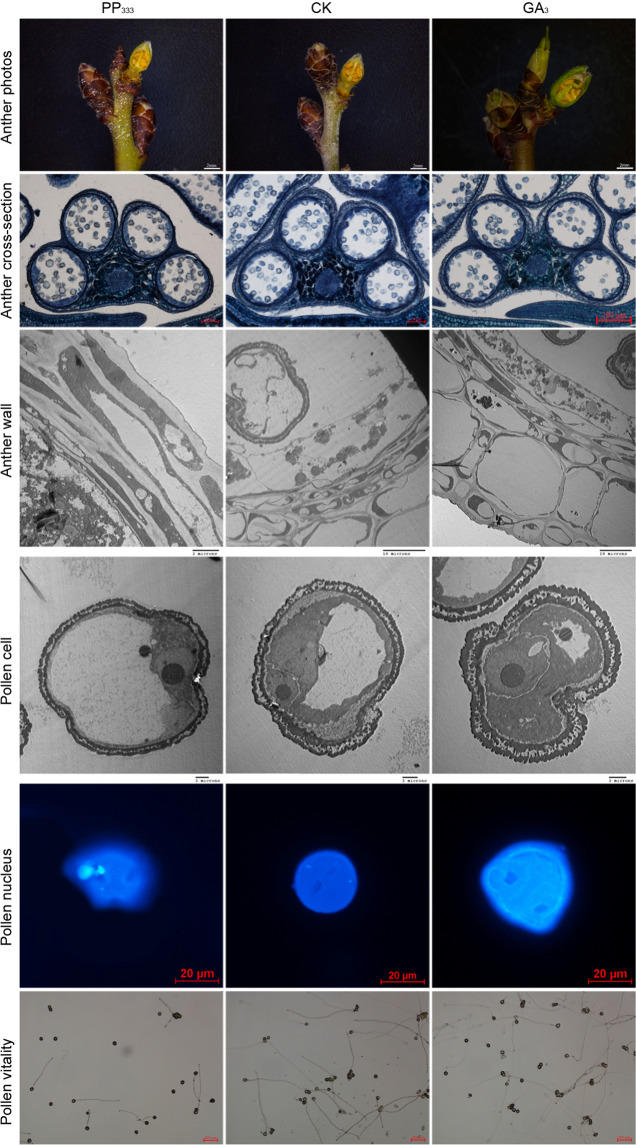
Function of GA in pollen development. A total of 600 branches from three trees at the ZP3 stage (1-nucleus stage) were treated at −12 °C until the relative sensitivity of conductivity in the buds reached approximately 50%, after which they were placed in a 4 °C climatic cabinet for 24 h. The treated samples were equally divided into three groups: a 100 μmol/L GA_3_-treated group and a CK group and a 100 μmol/L PP_333_-treated group. The branches were fixed in flower putty containing 1/2 MS+5% sucrose hydroponics in a climatic cabinet for 3 days. The other branches were cultivated to bloom in a climatic cabinet; their mature anthers were collected to test pollen viability. The anthers were photographed using a camera; the sections were photographed using microscopes; the anther wall and pollen cells were observed using SEM; the pollen nuclei were stained with DAPI and photographed under a microscope, and pollen viability was observed by microscopy

To understand the function of GA in almond anther development, we treated almond flowers with bioactive GA, GA_3_, and the GA biosynthesis inhibitor paclobutrazol (PP_333_). The results indicated that the growth rate per 1000 anthers in the water control group (CK) sample was 2.16 mg/d, which was higher than that in the PP_333_-treated sample (1.88 mg/d) and lower than that in the GA_3_-treated sample (2.74 mg/d) (Table 1). The sectioning results showed that these anthers were all in the 2-nucleus stage and exhibited 4 types of cells, including epidermal cells, endothelial cells, middle-layer cells, and tapetal cells. To further explore the differences among these three treatments, we used scanning electron microscopy to observe the anther microstructure. We found that epidermal cells, endothelial cells, and middle-layer cells in PP_333_-treated anthers were thinner than those in CK anthers, whereas these layers were the thickest in GA_3_-treated anthers. The tapetal cells were secretory and began to be degraded in CK anthers; they were degraded faster in GA_3_-treated anthers and more slowly in PP_333_-treated anthers. Furthermore, the microspores contained more fat droplets and starch granules and exhibited a richer sporopollenin cover in GA_3_-treated anthers than in the other two sample groups. The cytoplasm and nucleus of tapetal cells in PP_333_-treated anthers showed less degradation than those in CK and GA_3_-treated anthers. The pollen from PP_333_-treated anthers exhibited two nuclei, the largest vacuoles, small numbers of fat droplets and starch granules, and little sporopollenin cover. The pollen from CK anthers exhibited two nuclei, large vacuoles, some fat droplets and starch granules, and some sporopollenin cover. The pollen from GA_3_-treated anthers exhibited two nuclei, small vacuoles, many fat droplets and starch granules, and greater sporopollenin cover (Fig. 2).

To determine whether GA affected pollen viability, we sampled mature pollen from samples subjected to the three treatments to perform a germination assay. In the PP_333_-treated anthers, 42.00% of the pollen germinated, which was significantly lower than the percentage in CK anthers (49.33%); the pollen germination rate was also higher in the GA_3_-treated anthers (67.66%). Moreover, the number of pollen grains per anther was 1465 in the PP_333_-treated anthers, 1624 in the CK anthers, and 1804 in the GA_3_-treated anthers (Table 3). The results suggested that GA plays a role in increasing pollen quantity and vigor in almonds.

**Table 3:** Pollen quantity per anther and pollen viability

	Pollen quantity per anther	Pollen viability (%)
ZP4	2025 ± 79 Aa	73.93 ± 1.56 Aa
GA_3_-treated	1804 ± 22 Bb	67.66 ± 1.04 Bb
CK	1624 ± 39 Cc	49.33 ± 0.76 Cc
PP_333_-treated	1465 ± 44 Dd	42.00 ± 0.87 Dd

Different letters indicate significant differences between treatments or stages according to one-way ANOVA at *P* < 0.01 (capital letter) and *P* < 0.05 (lowercase letter)

In the hormone assays, we found that the levels of IAA, *t*Z, and JA were higher in GA_3_-treated anthers than in CK anthers, whereas there was no significant difference between PP_333_-treated and CK anthers. In contrast, the levels of ABA and SA were decreased in the GA_3_-treated group but exhibited no significant difference between the PP_333_-treated and CK groups (Table 2). These results regarding hormone levels combined with the phenotypes of anther development suggested that GA may regulate anther development by mediating the levels of different hormones.

### Analyses of transcriptome dynamics and module-trait relationships

**Fig. 3:**
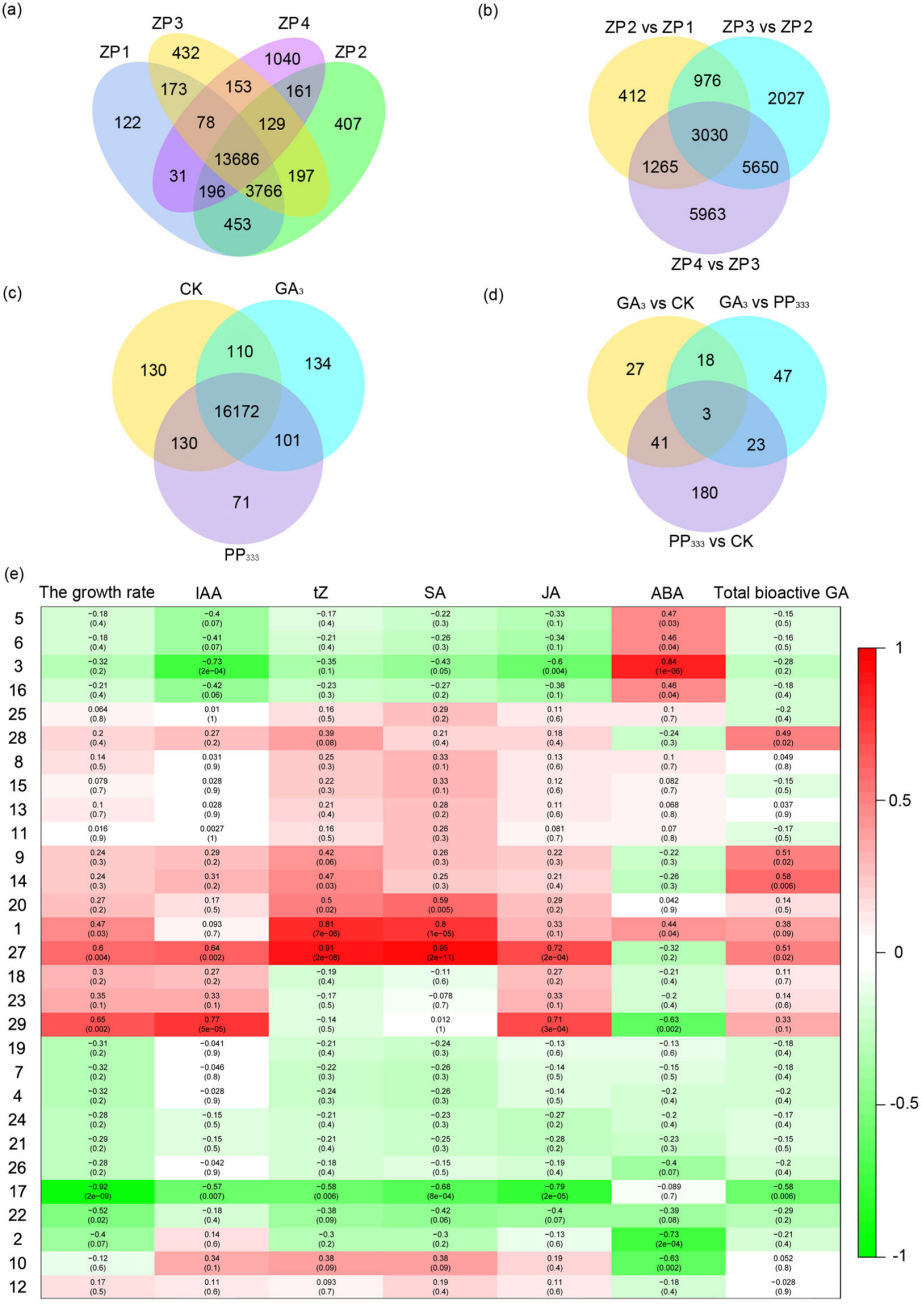
Differentially expressed unigenes in anthers and the module–trait relationships among genes, phytohormone levels, and growth rate per 1000 anthers. **a** Venn diagram of differentially expressed genes in the anthers of the four field samples. **b** Venn diagram of coexpressed genes in the anthers of the four field samples. **c** Venn diagram of differentially expressed genes in the anthers of the three treated samples. **d** Venn diagram of coexpressed genes in the anthers of the three treated samples. **e** Module-trait relationships among the genes, phytohormone levels, and growth rate per 1000 anthers

To study unigene dynamics and identify the candidate genes in anthers affected by exogenous GA_3_ and its inhibitor PP_333_, mRNA from four field samples and three treated samples at ZP3 (1-nucleus pollen stage) was sequenced. A total of 21,024 genes were detected in the 4 field samples, 13,686 of which were coexpressed (Fig. 3a), and 3030 differentially expressed genes (DEGs) were identified among them (Fig. 3b).

In the 3 treated samples, a total of 16,848 genes, including 16,172 coexpressed genes, were detected (Fig. 3c). Among these genes, 89 genes were regulated by exogenous GA_3_, and 91 genes were affected by PP_333_ (Fig. 3d). To explore the key genes involved in GA-regulated anther development in almond, we analyzed the module-trait relationships among the gene fragments per kilobase million (FPKM) values, phytohormone levels, and growth rate per 1000 anthers. The results indicated that the growth rate per 1,000 anthers exhibited a significant positive correlation with the gene FPKM values in modules 1, 27, and 29 but a significant negative correlation with the gene FPKM values in modules 17 and 22 at the 0.05 level (Fig. 3e).

### Analysis of reprogramming genes in anthers

**Fig. 4:**
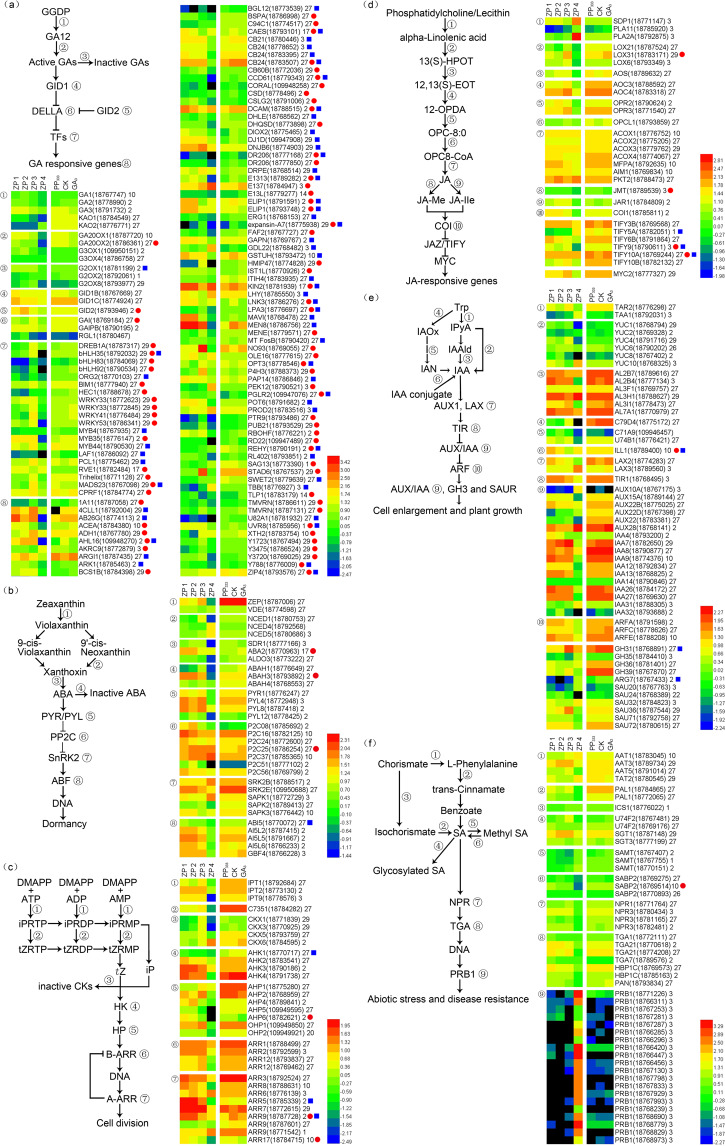
Heatmap of representative genes in six phytohormone pathways. **a** GA biosynthesis and signal transduction; **b** ABA biosynthesis and signal transduction; **c** *t*Z biosynthesis and signal transduction; **d** JA biosynthesis and signal transduction; **e** IAA biosynthesis and signal transduction; and **f** SA biosynthesis and signal transduction. Genes with red circles showed significant changes after GA_3_ spraying, while genes with blue squares showed significant changes after PP_333_ spraying. The module number is given after the gene name and gene ID

In GA biosynthesis and signal transduction, GA_12_ is the first plant gibberellin converted from trans-geranylgeranyl diphosphate (GGDP) by the ent-copalyl diphosphate synthase/GA requiring 1 (CPS/GA1), ent-kaur-16-ene synthase/GA requiring 2 (KS/GA2), ent-kaurene oxidase/GA requiring 3 (KO/GA3), and ent-kaurenoic acid oxidase (KAO) enzymes^14^. The gibberellin 20 oxidase (GA20OX) and gibberellin 3-beta-dioxygenase (GA3OX) enzymes promote the conversion of GA_12_ to bioactive GAs, and gibberellin 2-beta-dioxygenase (GA2OX) enzymes catalyze the 2-beta-hydroxylation of bioactive GA^28^. In the cytoplasm, the GA-GID1-SLR1 complex can be degraded through the participation of Gibberellin-Insensitive Dwarf 2 (GID2), leading to changes in transcription factors (TFs) and GA-responsive gene expression^29^. In this experiment, the content of total bioactive GA compounds showed a significant positive correlation with the gene FPKM values in modules 9, 14, 27, and 28 and a significant negative correlation with the gene FPKM values in module 17 (Fig. 3e). *GA20OX2* in module 27 and *GID2* in module 2 were downregulated, while the DELLA protein gene *gibberellin-insensitive* (*GAI*) in module 27 was upregulated, after treatment with exogenous GA_3_; *GA2OX2* in module 2 was downregulated by PP_333_. There were 20 TFs that were affected by exogenous GA_3_. *Dehydration-responsive element-binding protein 1A* (*DREB1A*), *basic helix–loop–helix transcription factors* (*bHLH35*, *bHLH83*, *bHLH92*, *BIM1*, *HEC1)*, *WRKY transcription factors* (*WRKY33*, *WRKY41*, and *WRKY53*), *MYB transcription factor 35* (*MYB35*), *trihelix*, *MADS-box transcription factor 23* (*MADS23*), and *common plant regulatory factor 1* (*CPRF1*) were upregulated by exogenous GA_3_, while *Reveille 1* (*RVE1*) was downregulated by exogenous GA_3_. Module-trait relationships showed that the FPKM values of *MYB4, MYB44*, *bHLH35*, *organ weight 2* (*bHLH38/ORG2*), *bHLH92*, *HEC1*, *WRKY33*, *WRKY41*, and *WRKY53* were obviously positively correlated with the growth rate per 1000 anthers, while the FPKM value of *RVE1* was obviously negatively correlated with the growth rate per 1000 anthers. An additional 84 genes were affected by either exogenous GA_3_ or PP_333_ (Fig. 4a).

ABA is converted from zeaxanthin and deactivated by ABA 8′-hydroxylase (ABAH)^14^. In the ABA signaling pathway, ABA is perceived by the ABA receptor pyrabactin resistance 1 (PYR1)/PYR1-like (PYL) proteins, which then deliver the signal to phosphatase 2C (PP2C) proteins^30^. PP2C proteins are negative regulators of the ABA pathway that prevent sucrose nonfermenting 1 (SNF1)-related protein kinase 2 (SnRK2) from activating downstream signals, such as ABA-responsive element-binding factor^31^. In this experiment, the ABA content showed a significant positive correlation with the gene FPKM values in modules 1, 3, 5, 6, and 16 and a significant negative correlation with the gene FPKM values in modules 2, 10, and 29 (Fig. 3e). *Short-chain dehydrogenase reductase 1*/*ABA-deficient 2* (*SDR1/ABA2*) in module 3 was downregulated by exogenous GA_3_; *ABAH1* and *phosphatase 2C 25* (*P2C25*) in module 27 were upregulated by exogenous GA_3_, and *abscisic acid-insensitive 5* (*ABI5*) in module 27 was downregulated by PP_333_ (Fig. 4b).

In *t*Z biosynthesis and signal transduction, IPT and cytokinin hydroxylase (C735A) promote the synthesis of *trans*-zeatin riboside monophosphate (*t*ZRMP) from dimethyl diphosphate (DMAPP) together with ATP, ADP, and AMP^32^. *t*ZRMP is converted to *t*Z with the participation of phosphatase and glycosidase, *t*Z is in turn deactivated by cytokinin oxidase (CKX), and a UDP-glycosyltransferase (UGT) superfamily protein bound to a soluble receptor histidine kinase (HK) transfers a phosphate group to a histidine-containing phosphotransferase (HP) protein and a two-component response regulator ARR protein to regulate cell division^14^. In this experiment, the *t*Z content showed a significant positive correlation with the gene FPKM values in modules 1, 14, 20, and 27 and a significant negative correlation with the gene FPKM values in module 17 (Fig. 3e). *AHP6* and *ARR9* in module 2 and *ARR17* in module 10 were downregulated by exogenous GA_3_ (Fig. 4c).

In JA biosynthesis and signal transduction, JA synthesis from lecithin is carried out by seven enzymes, with lipoxygenase (LOX) in the chloroplast being the rate-limiting enzyme in this process^14^. JA can be transformed to methyl jasmonate (MeJA) via the action of jasmonate O-methyltransferase (JMT) and to jasmonoyl-l-isoleucine (JA-Ile) via the action of JA-amido synthetase (JAR1)^33^. JA-Ile can disrupt TIFY domain/jasmonate-zim-domain (TIFY/JAZ) proteins together with a coronatine-insensitive (COI) protein and MYC2 to regulate JA-responsive genes^34^. In this experiment, the JA content showed a significant positive correlation with the gene FPKM values in modules 27 and 29 and a significant negative correlation with the gene FPKM values in modules 3 and 17 (Fig. 3e). *LOX31* in module 29, *TIFY9* in module 3, and *TIFY10A* in module 27 were upregulated by exogenous GA_3_, whereas *JMT* in module 3 was downregulated by exogenous GA_3_. *TIFY5A* in module 1, *TIFY9* in module 3, and *TIFY10A* in module 27 were upregulated by PP_333_ (Fig. 4d).

In IAA biosynthesis and signal transduction, IAA is synthesized from tryptophan (Trp) in two parallel pathways and can release free IAA conjugates under the action of IAA-amino acid hydrolase ILR1-like 1 (ILL1)^35^. IAA is transported by auxin-responsive protein 1 (AUX1) and auxin transporter-like (LAX) proteins^36^ and is perceived by transport inhibitor response (TIR) proteins^37^, which can regulate the expression of IAA-responsive genes, including the *auxin response factor* (*ARF*), *AUX/IAA*, *indole-3-acetic acid-amido synthetase GH3* and *SMALL AUXIN UPREGULATED RNA* (*SAUR*) genes, to further influence cell enlargement and plant growth^14^. In this experiment, the IAA content was significantly positively correlated with the gene FPKM values in modules 27 and 29 and significantly negatively correlated with the gene FPKM values in modules 3 and 17 (Fig. 3e). *ILL1* in module 10 was upregulated by exogenous GA_3_ and PP_333_, whereas *indole-3-acetic acid-amido synthetase GH3.1* (*GH3.1*) in module 27 and *indole-3-acetic acid-induced protein ARG7* (*ARG7*) in module 2 were downregulated by PP_333_ (Fig. 4e).

In SA biosynthesis and signal transduction, SA is synthesized from chorismate via two parallel pathways, in which the isochorismate synthase (ICS) pathway might be the main pathway^38^. There are several ways to reduce SA levels. UDP-glycosyltransferase 74F (U74F/SAG) transfers glucose to SA, forming a glucoside (SAG)^39^, and salicylate carboxymethyltransferase (SAMT) catalyzes the biosynthesis of methyl SA, which can be converted by SA-binding protein (SABP). *Nonexpressor of pr genes 1* (*NPR1*) is the key regulator of SA-JA crosstalk, mediates the binding of bZIP transcription factor family (TGA) members to the pathogenesis-related basic form of pathogenesis-related protein 1 (PRB1), and induces systemic acquired resistance (SAR) and defenses against abiotic stresses and disease^40^. In this experiment, the SA content exhibited a significant positive correlation with the gene FPKM values in modules 1, 20, and 27 and a significant negative correlation with the gene FPKM values in modules 3 and 17 (Fig. 3e). Only *SABP2* in module 10 was downregulated by exogenous GA_3_ (Fig. 4f).

### Validation of RNA-seq data

**Fig. 5:**
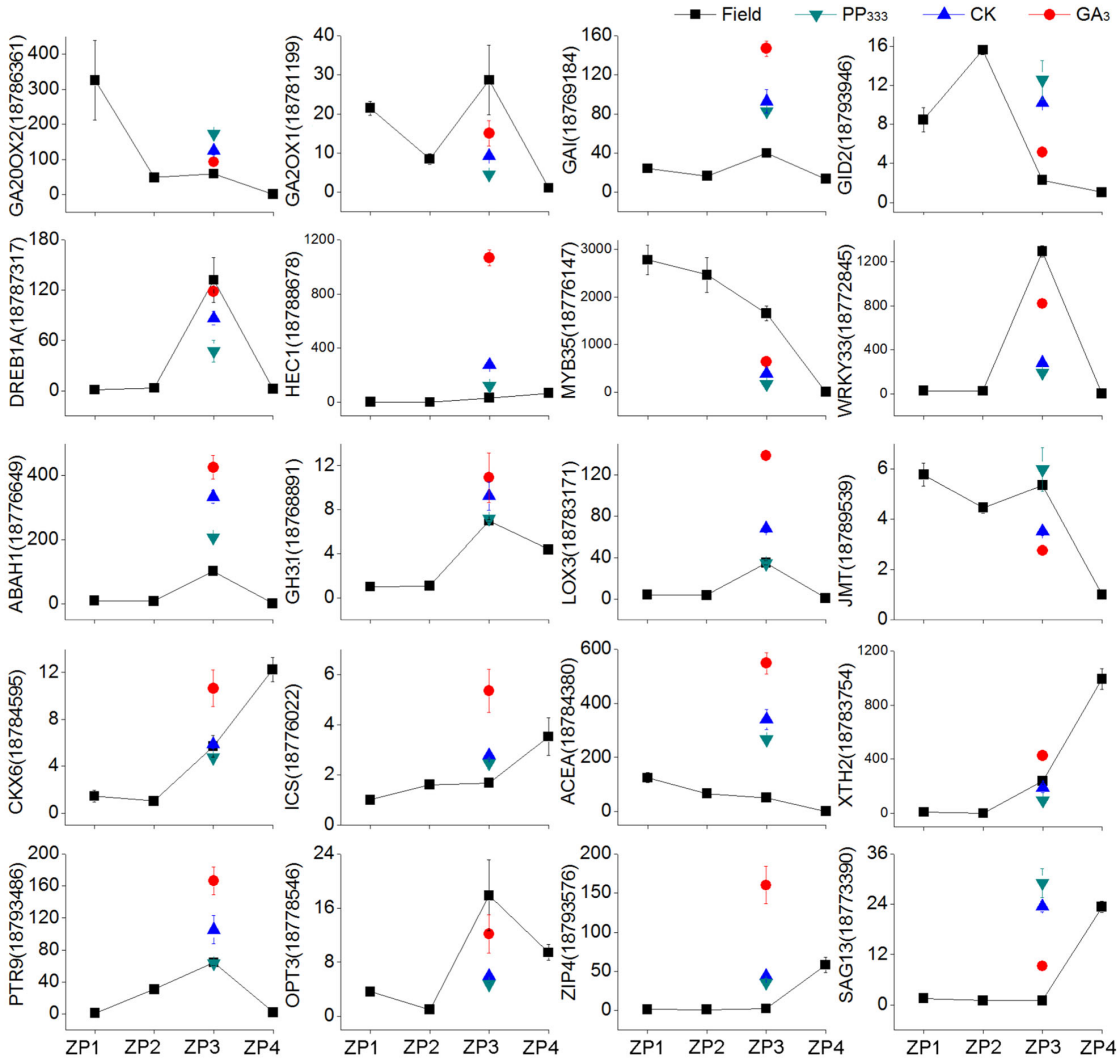
Validation of the expression levels of 20 unigenes. Relative expression levels of 20 hormone-related genes were tested by qRT-PCR method to confirm the RNA-seq data. The samples include field samples (ZP1, ZP2, ZP3, and ZP4) and treated samples (PP_333_ -treated, GA_3_ -treated, and CK). The values represent the means ± SD of three biological replicates

To evaluate the concordance of the gene expression intensities between RNA-seq and qRT-PCR, 20 unigenes related to GA_3_ treatment or PP_333_ treatment were selected to perform qRT-PCR to confirm the RNA-Seq data; moreover, the correlations of the changes in 20 unigenes between them were also investigated. High expression correlations, with high correlation coefficients (*R*^2^ > 0.75), were observed between the RNA-seq and qRT-PCR data (Fig. 5, Table S2). Most importantly, the expression levels of these selected genes determined by qRT-PCR were basically consistent with the RNA-seq results (Fig. 5, Table S2).

### Regulatory network of GA affecting anther development

**Fig. 6:**
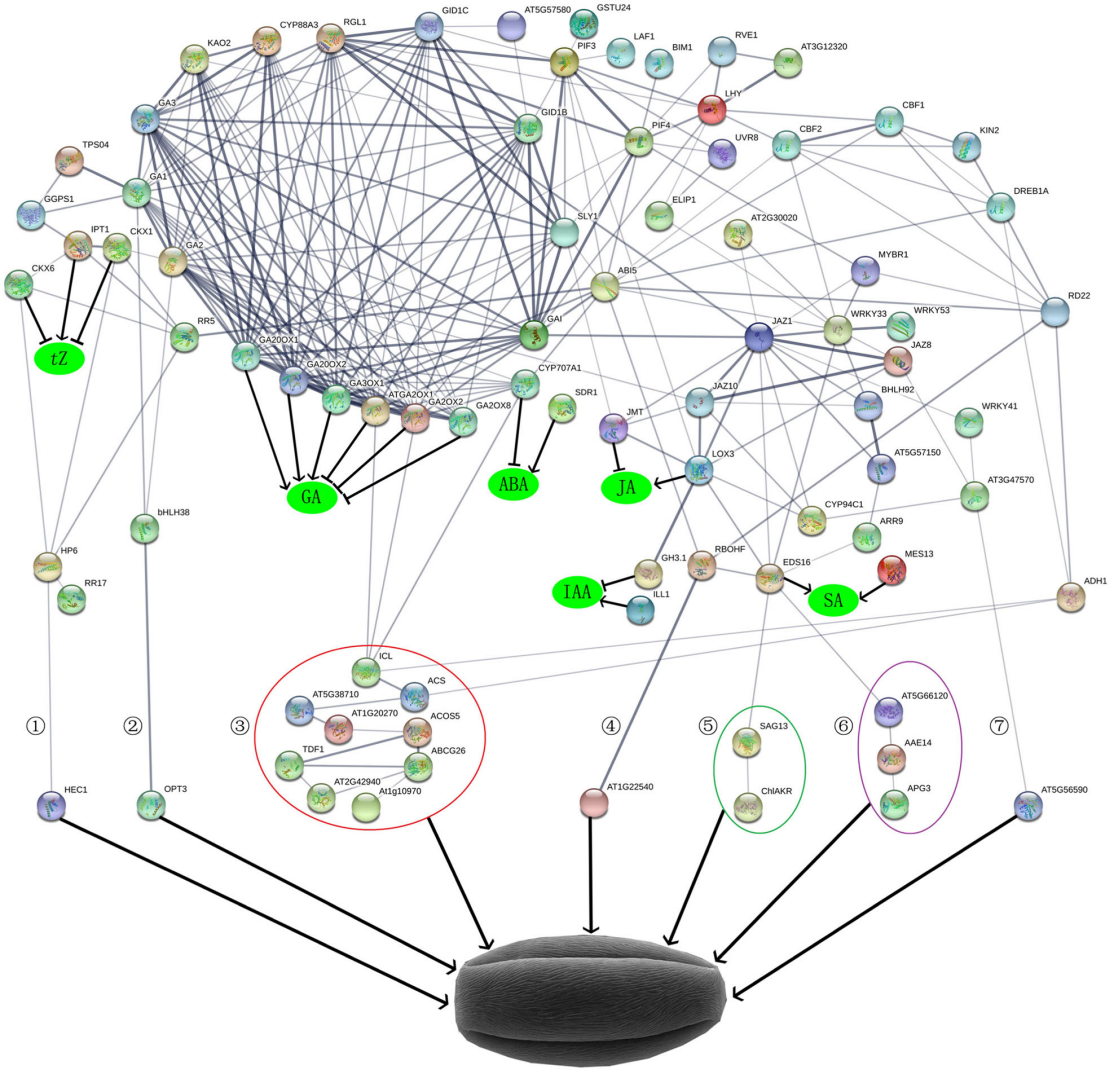
Regulatory network of GA in the development of almond pollen. ACOS5 = 4CLL1; AEE14 = MENE; ALDH11A3 = GAPN; APG3 = CPRF1; At1g10970 = ZIP4; At1G20270 = P4H3; AT1G22540 = PTR9; At2g30020 = P2C25; AT5G38710 = PROD2; AT2G42940 = AHL16; At3g47570 = Y3475; At5g56590 = E1313; AT5G57150 = bHLH35; AT5G66120 = DHQSD; CASP3 = CSD; ChlAKR = AKR4C9; CYP707A1 = ABAH1; EDS16 = ICS1; MES16 = SABP2; ICL = ACEA; MYBR1 = MYB44; TDF1 = MYB35

To elucidate the regulatory network of GA in the regulation of anther development, all DEGs related to the biosynthesis and signal transduction of six phytohormone pathways were selected based on the module–trait relationships of the gene FPKM values, phytohormone levels, and growth rate per 1000 anthers. The regulatory network was constructed by referring to the known protein–protein interactions in *Arabidopsis*. The regulatory network showed that GA regulated almond anther development by interacting with five phytohormone pathways and the DREB1A-mediated signaling pathway, which included the phytohormone biosynthesis and signal transduction genes *GA20ox2*, *GID2*, *GAI*, *SDR1*, *ABAH1*, *P2C25*, *ABI5*, *ARR5*, *ARR9*, *ARR17*, *AHK1*, *AHP6*, *ILL1*, *CH3.1*, *ARG7*, *LOX3*, *JMT*, *ADH1*, *TIFY5*, *TIFY9*, *TIFY10A*, *DIOX2*, *SABP2*, and *1-aminocyclopropane-1-carboxylate synthase 1* (*1A11/ACS*); the transcription factor genes *DREB1A*, *MYB35*, *MYB44*, *RVE1*, *bHLH35*, *HEC1*, and *WRKY33*; and the GA-responsive genes *oligopeptide transporter 3* (*OPT3*), *zinc transporter 4* (*ZIP4*), *ABC9 transporter G family member 26* (*ABCG26*), *4-coumarate-CoA ligase-like 1* (*4CLL1*), *protein NRT1/PTR FAMILY 5.14* (*PTR9*), *senescence-associated gene 13* (*SAG13*), *kinase 2* (*KIN2*), and *responsive to desiccation 22* (*RD22*), among others (Fig. 6).

## Discussion

GA is an important hormone involved in regulating anther development in plants^41^. A significantly reduced level of GA results in pollen sterility^42^. Moreover, GA plays an important role in cold responses during the reproductive stage in rice^43^. However, the function of GA in mediating pollen development in woody plants is less well studied. Therefore, the goal of this work was to determine whether and how GA functions in almond reproductive development. We analyzed microspore development and measured the hormone levels in the anthers at four pollen development stages. The results showed that the total bioactive GA compound levels gradually increased from ZP1 to ZP3 and that the correlation between the growth rate per 1000 anthers and the total bioactive GA compound content was 0.981, representing a significant correlation at the 0.05 level (two-sided). Our results indicate that bioactive GA compounds play important roles in almond anther development under normal- and low-temperature conditions.

### GA interacts with other hormones in almond anthers

Herein, we found that exogenous GA_3_ increased the levels of IAA, *t*Z, and JA and decreased the levels of SA and ABA. The balance of hormones is critical for pollen development and low-temperature responses; the balance between pollen development and cold tolerance is controlled by hormone homeostasis. IAA plays a role in the pollen production ability and fertility of spikelets. The increased level of IAA in almond in the GA treatment group helped to improve pollen vigor and fertility under normal- and low-temperature conditions. Cytokinin regulates anther development by coordinating carbon and nitrogen metabolism. In this study, we found that *t*Z, a kind of cytokinin, was induced by GA in almond anthers, showing that GA can stimulate the biosynthesis of cytokinins to regulate anther development. JA also affects flower development in plants, showing a higher level during anther development. The level of JA induced by GA contributes to improving pollen fertility under cold conditions. GA and ABA are antagonistic hormones, so the ABA level was reduced in GA-treated almond anthers in this study. However, JA can stimulate ABA overproduction to upregulate plant abiotic stress tolerance. Taken together, the results of our hormone analysis suggest that hormone balance regulated by GA is important for correct anther development.

We next sought to determine how GA regulates other hormones. Our RNA-seq data showed that GA affected the expression of many hormone biosynthesis-related genes. For example, the expression of the ABA biosynthesis gene *ABA2* was decreased, while the expression of the ABA-degradation gene *ABAH1* was increased, by exogenous GA_3_, indicating that GA reduces ABA levels. Moreover, GA_3_ induced the expression of *LOX3*, a key enzyme gene in JA synthesis, thereby increasing JA levels and contributing to anther development in almond. Furthermore, we found that signal transduction genes presented altered expression in the GA-treated group. These results imply that GA interacts with other hormones to regulate the expression of genes involved in their biosynthesis and thereby affects related signaling pathways.

### GA regulates the development of almond anthers

We found that exogenous GA_3_ promoted epidermal cell and endothecium cell elongation, tapetal cell degradation, and the accumulation of fat droplets and starch granules within pollen and sporopollenin on the pollen surface, while PP_333_ exerted the opposite effects. Exogenous GA_3_ increased the growth rate per 1000 anthers, pollen viability and the number of pollen grains per anther. To determine how GA regulated the development of almond anthers, we selected all the DEGs among the three treatment groups and constructed a regulatory network by referring to the known protein–protein interactions of *Arabidopsis*; we found that seven pathways might play a role in regulating the development of almond anthers (Fig. 6).

In the first pathway, the HEC1 protein showed an interaction with the AHP6 protein of the *t*Z pathway, and the FPKM value of *HEC1* in module 27 was upregulated by exogenous GA_3_ and positively correlated with the growth rate per 1000 anthers. A previous study showed that HEC1 might act as a local modulator of IAA and *t*Z to control gynoecium development^42^. Therefore, we propose that HEC1 might be a local modulator of the GA and *t*Z pathways to control anther development.

In the second pathway, the FPKM value of *bHLH38*/*ORG2* in module 27 was upregulated by PP_333_ and positively correlated with the growth rate per 1000 anthers, and the FPKM value of *OPT3* outside of the 29 modules was upregulated by both exogenous GA_3_ and PP_333_. Previous evidence has shown that bHLH38/ORG2 plays a role in Fe regulation^43^ and that OPT3 is involved in Fe, Zn, Cd, and oligopeptide translocation^44^. Therefore, we surmise that bHLH38/ORG2 and OPT3 function in anther development as nutrient transporters.

Based on the third pathway, the expressions of *ZIP4*, *4CLL1/ACOS5*, and *prolyl 4-hydroxylase 3 (P4H3)* were positively correlated with the growth rate per 1000 anthers. The expression of *ZIP4* and *1A11/ACS* was elevated by both GA_3_ and PP_333_; the levels of *MYB35* and *P4H3* were induced by exogenous GA_3_; and the expression of *ABCG26* and *4CLL1/ACOS5* was repressed by PP_333_. Evidence shows that MYB35/TDF1 is located downstream of dysfunctional tapetum 1 (DYT1) in the control of tapetal development and pollen wall formation^45^. ZIP4 is considered an important Zn uptake transporter^46^; ABCG26 plays a role in pollen exine formation by exporting polyketide traffic^47^; 4CLL1/ACOS5, which is mainly expressed in the tapetum, is required for pollen wall exine formation^48^; and P4H3 catalyzes the formation of 4-hydroxyproline for the construction of plant cell wall glycoproteins^49^. Therefore, nutrient transport, regulation of pollen exine formation and tapetum degradation, and ethylene biosynthesis are critical for anther development in almond.

In this study, the expression of *respiratory burst oxidase protein F* (*RBOHF*) in module 2 and *PTR9* in module 27 was increased by exogenous GA_3_ and was positively correlated with the growth rate per 1000 anthers. Previous studies have shown that *RBOHF* is induced by low temperature, drought, salt, ABA, and H_2_O_2_ and mediates diverse physiological processes^50^ and that PTR9 is involved in oligopeptide transport^51^. Thus, the available data indicate that GA regulates anther development by improving oligopeptide transport and stress responses.

Moreover, we found that *WRKY53, SAG13, DHQSD* (*bifunctional 3-dehydroquinate dehydratase/shikimate dehydrogenase*)*, MENE/AAE14*, and *CPRF1/APG3* levels were elevated by GA and that *aldo-keto reductase family 4 member C9* (*AKR4C9*) and *glucan endo-1,3-beta-glucosidase* (*E1313*) levels were reduced by GA. WRKY53 and SAG13 function in the regulation of senescence^52^. AAE14/MENE is involved in phylloquinone biosynthesis^53^; CPRF1*/*APG3 plays an important role in chloroplast development^54^, and DHQSD catalyzes the second step in the shikimate pathway^55^. E1313/At5g56590 is involved in the degradation of callose walls around the microspore tetrad and is essential for pollen exine formation^56^. Based on these data, we speculate that GA regulates anther tissue senescence by interacting with SA to affect anther development in almond.

### GA regulates the resistance of anthers to low temperature

In our experiment, some stress-responsive genes, including *DREB1A*, *bHLH92*, *WRKY41*, *KIN2*, and *ADH1*, were induced in the GA-treated groups. Most of these genes showed obvious positive correlations with the growth rate per 1000 anthers. Previous studies have revealed that *DREB1A* plays a crucial role in cold acclimation and freezing resistance by positively regulating DELLAs and thereby inducing the expression of *KIN2* and *RD22*^57^. ADH1 enhances the freezing resistance of plants^46^. bHLH92 functions in the osmotic stress response^58^. MYB4 is a negative regulator that improves stress tolerance^59^, and MYB44 and RVE1 can regulate stress resistance^60^. WRKY33 and WRKY41 regulate phytohormone levels and responses to biotic and abiotic stress^61,62^. Therefore, GA improves anther cold tolerance by regulating stress-responsive genes.

## Materials and methods

### Plants, treatment, and sampling

“Zhipi” almond growing in the Plant Resources Garden of the Xinjiang Academy of Agricultural Sciences (Luntai, Xinjiang, 41° 46′ 58′′ N, 84° 13′ 24′′ E) was selected as the study material. The following samples were collected from early winter to spring between 9:00 and 10:00 A.M.: ZP1 (“Zhipi” at the meiosis stage in the field, January 1), ZP2 (“Zhipi” at the tetrad stage in the field, February 1), ZP3 (“Zhipi” at the 1-nucleus stage in the field, March 1), and ZP4 (“Zhipi” at the mature 2-nucleus stage in the field, March 28). ZP3 flowers were more sensitive to cold and had higher bioactive GA levels than flowers of other stages, so ZP3 flower buds were chosen for further detailed experiments. ZP3 flower buds were treated with exogenous hormones, and 600 branches were cut from 3 trees on March 1, treated at −12 °C until the relative sensitivity of conductivity in the buds reached approximately 50%, and then maintained in a 4 °C climatic cabinet for 24 h. The treated samples were equally divided into three groups: a 100 μmol/L GA_3_-treated group, a CK group, and a 100 μmol/L PP_333_-treated group. The branches were fixed in flower putty containing 1/2 MS+5% sucrose hydroponic solutions in a climatic cabinet for 3 days; thereafter, the anthers were collected from 100 branches and stored in liquid nitrogen^28^. The other branches were cultivated to bloom in a climatic cabinet; their mature anthers were collected for pollen quantification and pollen viability assessment.

### Photographs and sections of anthers

Photographs of anthers were taken on a Nikon SMZ-250 stereomicroscope. Paraffin sections of anthers were dyed with toluidine blue, and photographs were taken on a Nikon 80i transmission microscope. Fifty-nanometer-thick anther sections were cut with an LKB-8800 ultrathin slicer, stained with uranyl acetate-lead citrate, and observed on an H-600 transmission electron microscope. Complete pollen samples were dyed with a 5% DAPI solution for 30 min in the dark and observed on a Nikon 80i transmission fluorescence microscope.

### Measurement of pollen indexes

Pollen was collected at the time of flowering, and the viability of 1000 pollen grains was tested on a medium containing 10% sucrose and 1% agar powder in a 25 °C incubator for 12 h. The number of pollen grains per anther was counted under a microscope^41^.

### Measurement of different phytohormones

Anthers (1.00 ± 0.05 g, stored in a −80 °C freezer) were accurately weighed and ground in liquid nitrogen; 10 ml of isopropanol-hydrochloric acid extraction buffer was added to the anther powder, and the mixture was shaken at 4 °C for 30 min. Then, 20 ml of dichloromethane was added to the mixtures, which were shaken at 4 °C for 30 min. The treated anthers were next centrifuged at 13,000 r/min for 5 min at 4 °C to obtain the lower organic phase, and the organic phase was dried with nitrogen in the dark, dissolved in 400 μl of methanol containing 0.1% formic acid, and filtered through a 0.22-μm filter to detect the levels of GA compounds, IAA, JA, ABA, *t*Z, and SA by HPLC-MS^41^.

### RNA isolation, sequencing, and qRT-PCR

Total RNA was isolated from almond anthers with Power SYBR^®^ Green PCR Master Mix (Applied Biosystems^®^ Cat: 4367659) and quantified using BIO-RAD CFX Connect^™^ (USA). The expression levels determined by qRT-PCR were analyzed based on the average mean of three biological repeats. The quantity of RNA in each sample was estimated with a spectrophotometer and by agarose gel electrophoresis, and samples with higher quality were selected for sequencing on the Illumina PE150 platform; the RNA-Seq data had three biological repetitions. Twenty genes were selected to design primers (Table S3) and validate the RNA-seq data (Table S2), and the *AcActin1* gene was used as an internal standard for the normalization of data to calculate relative fold differences based on the comparative cycle threshold (2^−ΔΔCT^) values^4^. All the experiments in this study had more than three biological replicates.

### Module–trait relationship map, heatmap, and protein interaction map

A module–trait relationship map was produced by weighted correlation network analysis based on the FPKM values of the genes, phytohormone levels and growth rate per 1000 anthers. If one variable moved proportional to another variable, there was a positive correlation (*r* > 0); in contrast, if variables opposed one another, they displayed a negative correlation (*r* < 0). The heatmaps of the genes were generated with HemI 1.0 based on the FPKM values of the genes and were refined with Photoshop CS5. The protein-protein interaction maps were produced with the STRING database by referring to the data for *Arabidopsis thaliana*.

## Supplementary information

supporting data
