# Supplementary material for: Interaction of gibberellin and other hormones in almond anthers: phenotypic and physiological changes and transcriptomic reprogramming
Source: Hortic Res. 2021 May 1;8:94. doi: 10.1038/s41438-021-00527-w (PMC8087710; doi:10.1038/s41438-021-00527-w)
Supplement: Supplementary file 1 — supporting data [file 41438_2021_527_MOESM1_ESM.docx]

Supporting information

**Table S1** Correlation between the growth rate per 1,000 anthers and hormones

**Table S2** Correlation between the qPCR expression and RNA-seq data

**Table S3** The primers used in this study

Table S1 Correlation between the growth rate per 1,000 anthers and hormones

|  | Rate of growth | IAA | *t*Z | SA | JA | ABA | Bioactive GA |
| --- | --- | --- | --- | --- | --- | --- | --- |
| Rate of growth | 1 |  |  |  |  |  |  |
| IAA | 0.773 | 1 |  |  |  |  |  |
| tZ | 0.622 | 0.946 | 1 |  |  |  |  |
| SA | 0.919 | 0.956^*^ | 0.876 | 1 |  |  |  |
| JA | 0.899 | 0.962^*^ | 0.835 | 0.981^*^ | 1 |  |  |
| ABA | -0.26 | -0.771 | -0.917 | -0.612 | -0.57 | 1 |  |
| Bioactive GA | 0.988^*^ | 0.848 | 0.733 | 0.965^*^ | 0.936 | -0.402 | 1 |

Note: * is significantly correlated at a 0.05 level (both sides).

Table S2 Correlation between the qPCR expression and RNA-seq data

|  | FPKM | | | | | | | qRT-PCR | | | | | | |  |
| --- | --- | --- | --- | --- | --- | --- | --- | --- | --- | --- | --- | --- | --- | --- | --- |
|  | ZP1 | ZP2 | ZP3 | ZP4 | GA_3_ | CK | PP_333_ | ZP1 | ZP2 | ZP3 | ZP4 | GA_3_ | CK | PP_333_ | R^2^ |
| AcDREB1A | 0.9 | 1.8 | 35.5 | 3.1 | 12.4 | 7.9 | 5.7 | 1.0 | 3.0 | 132.2 | 2.2 | 118.7 | 86.7 | 47.3 | 0.83 |
| AcGA20OX2 | 15.8 | 5.7 | 3.2 | 0.0 | 15.8 | 26.2 | 29.3 | 325.4 | 48.7 | 59.2 | 1.0 | 92.6 | 124.8 | 172.3 | 0.94 |
| AcGA2OX1 | 12.6 | 16.8 | 18.9 | 1.4 | 6.5 | 6.0 | 4.6 | 21.5 | 8.4 | 28.7 | 1.0 | 15.0 | 9.2 | 4.5 | 0.78 |
| AcGID2 | 89.6 | 159.6 | 26.8 | 8.0 | 31.7 | 50.5 | 49.4 | 8.5 | 15.6 | 2.3 | 1.0 | 5.1 | 10.2 | 12.6 | 0.83 |
| AcGAI | 15.7 | 7.4 | 23.1 | 3.4 | 47.3 | 25.8 | 31.8 | 21.6 | 16.4 | 49.3 | 13.4 | 146.9 | 92.9 | 82.5 | 0.86 |
| AcLOX31 | 14.1 | 9.6 | 98.0 | 1.1 | 61.8 | 39.6 | 42.4 | 4.3 | 3.7 | 35.0 | 1.0 | 138.4 | 68.0 | 34.3 | 0.81 |
| AcJMT | 2.5 | 1.9 | 2.5 | 0.4 | 1.5 | 2.8 | 3.6 | 5.8 | 4.5 | 5.3 | 1.0 | 2.8 | 3.5 | 6.0 | 0.90 |
| AcGH31 | 19.7 | 15.2 | 182.6 | 34.9 | 132.0 | 126.4 | 84.7 | 1.0 | 1.1 | 7.0 | 4.4 | 10.9 | 9.2 | 7.2 | 0.86 |
| AcABAH1 | 0.4 | 0.6 | 8.7 | 0.1 | 11.1 | 6.3 | 5.7 | 9.6 | 8.8 | 102.2 | 1.0 | 425.6 | 333.9 | 206.8 | 0.95 |
| CKX6 | 1.5 | 0.9 | 4.6 | 31.1 | 10.3 | 8.3 | 8.3 | 1.5 | 1.0 | 5.7 | 12.3 | 10.6 | 5.9 | 4.7 | 0.91 |
| AcICS | 1.7 | 2.2 | 3.4 | 4.5 | 6.9 | 5.6 | 5.6 | 1.0 | 1.6 | 1.7 | 3.5 | 5.4 | 2.8 | 2.5 | 0.82 |
| AcMYB35 | 121.4 | 78.6 | 86.4 | 0.0 | 31.3 | 23.5 | 18.4 | 2778.8 | 2465.0 | 1652.8 | 1.0 | 637.6 | 387.6 | 167.7 | 0.97 |
| AcWRKY33 | 1.5 | 6.0 | 51.9 | 0.5 | 30.8 | 15.1 | 13.1 | 27.1 | 25.2 | 1295.5 | 1.0 | 820.4 | 281.9 | 190.3 | 0.93 |
| AcHEC1 | 0.1 | 0.1 | 1.0 | 1.0 | 9.1 | 3.6 | 3.7 | 1.7 | 1.0 | 32.5 | 67.8 | 1068.3 | 276.7 | 121.2 | 0.98 |
| AcXTH2 | 1.1 | 0.1 | 52.9 | 33.1 | 26.8 | 15.7 | 12.3 | 9.3 | 1.0 | 239.4 | 895.2 | 424.9 | 189.9 | 96.8 | 0.94 |
| AcACEA | 29.2 | 21.1 | 7.4 | 0.1 | 22.3 | 13.9 | 17.4 | 124.9 | 66.2 | 51.0 | 1.0 | 548.4 | 340.5 | 267.1 | 0.85 |
| AcPTR9 | 0.01 | 0.1 | 0.8 | 0.0 | 2.1 | 0.8 | 0.9 | 1.1 | 38.4 | 64.3 | 1.8 | 166.6 | 105.4 | 63.4 | 0.89 |
| AcOPT3 | 7.91 | 2.9 | 40.8 | 15.0 | 23.7 | 14.9 | 27.9 | 3.7 | 1.0 | 17.9 | 9.4 | 12.2 | 6.0 | 4.8 | 0.81 |
| AcZIP4 | 18.3 | 10.2 | 28.3 | 33.3 | 76.9 | 44.9 | 71.7 | 1.1 | 1.0 | 2.1 | 58.1 | 160.1 | 43.8 | 35.0 | 0.76 |
| AcSAG13 | 1.7 | 0.9 | 1.6 | 12.0 | 18.5 | 31.9 | 27.0 | 1.5 | 1.0 | 1.0 | 23.4 | 9.2 | 23.5 | 29.1 | 0.93 |

Table S3 The primers used in this study

| name | gene code | direction | sequence（5’-3’） | Tm (℃) | Size (bp) |
| --- | --- | --- | --- | --- | --- |
| Actin | AM491134.1 | F | TCCTGAAGAGCACCCAGTTC | 59 | 116 |
|  |  | R | TGGCAACATACATAGCAGGC | 58 |  |
| AcTubulin | X67162.1 | F | ATCCCTCTCCACAGGTCTCT | 59 | 143 |
|  |  | R | AATGTCAAGAGAACGCCTGC | 59 |  |
| AcDREB 1A | 18787317 | F | TGCTTGAACTTTGCCGACTC | 59 | 180 |
|  |  | R | TTGTTCCGATGCTGACTTGC | 59 |  |
| AcGA20OX2 | 18786361 | F | AATTACTACCCACCGTGCCA | 59 | 117 |
|  |  | R | CACAAACACCTCAAGCCCTC | 59 |  |
| AcGA2OX1 | 18781199 | F | CTTCTCCTTGCCGCTTTCTG | 59 | 104 |
|  |  | R | ACTCCACCCAACCAACATCA | 59 |  |
| AcGID2 | 18793946 | F | CATCTCCGTTGAAGCCCATG | 59 | 131 |
|  |  | R | TCATCTGCCTCTGTTAGCGT | 59 |  |
| AcGAI | 18769184 | F | CGCTGGTTACTTCATCGACG | 59 | 142 |
|  |  | R | CTGATTGGCGGTGAAGTGAG | 59 |  |
| AcLOX31 | 18783171 | F | GCACCCAAGAAAAGCAGTGA | 59 | 132 |
|  |  | R | AACTGTGATTGCTCCTGGGA | 59 |  |
| AcJMT | 18789539 | F | GTCGTTCGTAATGGACCGTG | 59 | 130 |
|  |  | R | TAGCATTGACTCAGCCACGA | 59 |  |
| AcGH31 | 18768891 | F | CCGCTAGAGATACGTGTGGT | 59 | 110 |
|  |  | R | AAATTCACACACCTCGGCAC | 59 |  |
| AcABAH1 | 18776649 | F | TCCAGGCACACTCTTTCACA | 59 | 147 |
|  |  | R | AGGCCTTCTTTGTCACCCAT | 59 |  |
| AcCKX6 | 18784595 | F | CGGTCTGCTAAACAACTGGAG | 59 | 119 |
|  |  | R | CCAGGTTGAAGTATTTGGCCA | 58 |  |
| AcICS | 18776022 | F | AGAGGAGGCACGGGTTTTAA | 59 | 105 |
|  |  | R | CCAACGGCAAACTCACTCTC | 59 |  |
| AcMYB35 | 18776147 | F | TACTTGTTCATCTGCTGCGC | 59 | 131 |
|  |  | R | TCGACGAATGCTCAGCCTTA | 59 |  |
| AcWRKY33 | 18772845 | F | TGAAGGGCAGTGAAAATCCG | 58 | 144 |
|  |  | R | TAGACTGAGGCTTGGGATGG | 59 |  |
| AcHEC1 | 18788678 | F | ATCGACCCAGAATCCGTGAA | 59 | 100 |
|  |  | R | CGCTGATCCTCTCTCTCCTG | 59 |  |
| AcXTH2 | 18783754 | F | AGCAGAGTGAATGGCAGACT | 59 | 139 |
|  |  | R | CGTCTGCATTCAACTGGGAG | 59 |  |
| AcACEA | 18784380 | F | TTTGAAGCCGAGGTAGCAGA | 59 | 105 |
|  |  | R | GGTCCCTCTTAGTGCCACAA | 59 |  |
| AcPTR9 | 18793486 | F | TGGCTGTGGAATGGCTTCTA | 59 | 102 |
|  |  | R | GTGTCATCTTCAAGCCCTGC | 59 |  |
| AcOPT3 | 18778546 | F | TGGTGGATGTTGGAGAGCAT | 59 | 139 |
|  |  | R | CAAACAGTCGCTTTGGTCCA | 59 |  |
| AcZIP4 | 18793576 | F | CGGAATGGGAAGGTGTTTGG | 59 | 147 |
|  |  | R | GTGTTCAAGTTCGGGTTCCC | 59 |  |
| AcSAG13 | 18773390 | F | CTGGCGTTGTATCAGTTGGG | 59 | 100 |
|  |  | R | TCTTTTGCCCACTCACATGC | 59 |  |
